# Genome Sequence of Strain MOLA814, a Proteorhodopsin-Containing Representative of the *Betaproteobacteria* Common in the Ocean

**DOI:** 10.1128/genomeA.01062-13

**Published:** 2013-12-19

**Authors:** Alicia Courties, Thomas Riedel, Michael Jarek, Laurent Intertaglia, Philippe Lebaron, Marcelino T. Suzuki

**Affiliations:** UPMC Université Paris 6, UMR 7621, Observatoire Océanologique, Banyuls-sur-Mer, Francea; CNRS, UMR 7621, LOMIC, Observatoire Océanologique, Banyuls-sur-Mer, Franceb; Helmholtz-Centre for Infection Research, Research Group Genomic Analytics, Braunschweig, Germanyc

## Abstract

Strain MOLA814 is a marine betaproteobacterium that was isolated from seawater in the Beaufort Sea. Here, we present its genome sequence and annotation. Genome analysis revealed the presence of a proteorhodopsin-encoding sequence together with its retinal-producing pathway, indicating that this strain might generate energy by using light.

## GENOME ANNOUNCEMENT

Marine strain MOLA814 was isolated from a depth of 3 m in the Canadian Beaufort Sea (71°40.294′N, 130°43.674′W). This strain belongs to the *Betaproteobacteria*, and its 16S rRNA sequence is 98% identical to that of strain OTU126, which is described as the 40th most abundant operational taxonomic unit (OTU) among >45,000 sequences from surface ocean planktonic prokaryotes (1).

The genomic DNA of strain MOLA814 was extracted using the cetyltrimethylammonium bromide (CTAB) protocol (2). Library preparation for whole-genome sequencing was performed using the TruSeq DNA PCR-free sample preparation kit (Illumina, San Diego, CA) with 550-bp insert sizes, according to the manufacturer’s protocol. Genomic DNA was shared using a Covaris S2 system (Covaris, Woburn, MA) and subjected to end repair, purification, and ligation of the fragments with multiple indexed adapters for library preparation. Quality control of the prepared library was validated using quantitative PCR (qPCR) (Kapa library quantification kit; Kapa Biosystems, Woburn, MA) and an Agilent Bioanalyzer high-sensitivity (HS) chip (Agilent Technologies, Santa Clara, CA) according to the manufacturers’ instructions. Genome sequencing was performed to 250 cycles in both directions in a MiSeq system (Ilumina), which generated 2,446,022 total reads (611.5 Mbp). DNA-Seq reads were converted to Fastq format and *de novo* assembled with Velvet 1.2.07 (3). The sequencing data were controlled for general quality features using the FastqMcf tool of ea-utils (http://code.google.com/p/ea-utils). The resulting 3 scaffolds with 87× average coverage of the genome were annotated using Prokka 1.7 (4).

The draft genome sequence of strain MOLA814 is 2,859,706 bp in size, contains 2,683 coding sequences, 3 rRNAs, and 39 tRNAs, and has a G+C content of 53.6%.

Interestingly, the genome analysis of strain MOLA814 revealed the presence of a proteorhodopsin-encoding gene sequence (PR) and a putative retinal-producing biosynthetic pathway (5–7). The PR-encoding sequence codes for a green light-absorbing PR-opsin (8, 9) of 263 amino acid residues with the typical features necessary for proton pump activity, like Asp97 and Glu108 residues (eBAC31A08 numbering). These act as proton acceptor and donor in the retinylidene Schiff base transfer during the PR photocycle. BLAST analysis (10) revealed high PR protein sequence identities to the PR sequences of representatives belonging to the alphaproteobacterial SAR116 clade, like “*Candidatus* Puniceispirillum marinum” IMCC1322 (11).

The presence of a PR-encoding sequence together with its retinal-producing pathway in the genome sequence indicates a putative photoheterotrophic lifestyle. In addition, the genome sequence of strain MOLA814 provides a good opportunity for studying the physiological and ecological functions of a commonly occurring marine betaproteobacterium living in ocean waters.

### Nucleotide sequence accession numbers.

The whole-genome shotgun project has been deposited at DDBJ/EMBL/GenBank under the accession no. AYMW00000000. The version described in this paper is version AYMW01000000.
